# Neurofeedback and biofeedback with 37 migraineurs: a clinical outcome study

**DOI:** 10.1186/1744-9081-6-9

**Published:** 2010-02-02

**Authors:** Deborah A Stokes, Martha S Lappin

**Affiliations:** 1 The Better Brain Center, Inc, 2121 Eisenhower Ave Suite 604 Alexandria, VA 22314, USA; 2 Action Research and Technical Solutions, Inc, 11506 Links Drive Reston, VA 20190, USA

## Abstract

**Background:**

Traditional peripheral biofeedback has grade A evidence for effectively treating migraines. Two newer forms of neurobiofeedback, EEG biofeedback and hemoencephalography biofeedback were combined with thermal handwarming biofeedback to treat 37 migraineurs in a clinical outpatient setting.

**Methods:**

37 migraine patients underwent an average of 40 neurofeedback sessions combined with thermal biofeedback in an outpatient biofeedback clinic. All patients were on at least one type of medication for migraine; preventive, abortive or rescue. Patients kept daily headache diaries a minimum of two weeks prior to treatment and throughout treatment showing symptom frequency, severity, duration and medications used. Treatments were conducted an average of three times weekly over an average span of 6 months. Headache diaries were examined after treatment and a formal interview was conducted. After an average of 14.5 months following treatment, a formal interview was conducted in order to ascertain duration of treatment effects.

**Results:**

Of the 37 migraine patients treated, 26 patients or 70% experienced at least a 50% reduction in the frequency of their headaches which was sustained on average 14.5 months after treatments were discontinued.

**Conclusions:**

All combined neuro and biofeedback interventions were effective in reducing the frequency of migraines with clients using medication resulting in a more favorable outcome (70% experiencing at least a 50% reduction in headaches) than just medications alone (50% experience a 50% reduction) and that the effect size of our study involving three different types of biofeedback for migraine (1.09) was more robust than effect size of combined studies on thermal biofeedback alone for migraine (.5). These non-invasive interventions may show promise for treating treatment-refractory migraine and for preventing the progression from episodic to chronic migraine.

## Background

Migraine is a common, disabling and often progressive disorder characterized by increased excitability of the central nervous system [1,2]. It occurs in 18% of women and 6% of men in the US with peak prevalence in individuals between the ages of 25 and 55 [3]. Economic burden of migraine in the US is estimated to be approximately 13 billion annually [4]. Biofeedback is a common intervention in pain management. For migraine treatment, the most frequently used biofeedback methods have been peripheral skin temperature biofeedback, blood- volume-pulse and electromyography feedback [5]. In a recent meta-analysis involving biofeedback for the treatment of migraine, Grade A evidence [6] was found for the efficacy of the above methods which proved stable over a 17 month follow-up phase [5]. Numerous studies explore peripheral biofeedback [5] but scant studies exist on using neurofeedback methods to treat migraine [7-11]. Although this study looks at neurofeedback, it is not the sole intervention. Instead of providing only neurofeedback protocols as the sole modality in this clinical setting, the lead author, who is a clinician in private practice, decided early on to utilize evidence-based thermal biofeedback methods in addition to the neurofeedback in order to maximize the patient's chances of success.

Neurotherapy is a broad term referring to the many types of biofeedback used to deliver information about the central nervous system which involve blood flow, thermal output from the brain or electrical activity. Neurofeedback (also called neurobiofeedback or EEG biofeedback) usually refers to frequency-based biofeedback that uses an EEG to give clients information about their brainwaves and gradually and subtly teaches people how to alter their brainwave activity. Sensors are attached to the scalp and the raw EEG signal is amplified, the frequency spectrum is extracted via a Fourier transform and selected frequency components are displayed through a user interface such as a video game. Unlike peripheral biofeedback that monitors the status of peripheral aspects of the sympathetic and parasympathetic nervous systems (e.g. respiration, galvanic skin response), neurofeedback monitors central nervous system activity.

A preliminary review of the literature on the clinical applications of neurofeedback suggests that it may be effective for a number of cognitive, emotional, and physical problems [12-15]. The neurofeedback literature is still in its infancy, and with the exception of ADHD, there are few double-blind, placebo-controlled studies. Although there are randomized controlled studies of neurofeedback applied to conditions such as ADD/ADHD, uncontrolled epilepsy, learning disabilities, anxiety, post traumatic stress disorder, alcoholism, autism and traumatic brain injury [15], there are many areas of application including migraine where controlled studies using neurofeedback do not yet exist. Nevertheless, the body of research is growing, and the practice-based studies that form the basis of much of the published literature are stimulating a great deal of interest in the field.

Abnormalities in electrophysiological activity have commonly been found in the brains of migraine patients [16-21], therefore it is plausible that interventions involving the EEG might be of benefit [16]. Children afflicted with migraine, those with and without aura, demonstrate increased theta frequencies compared to normal controls [17]. One popular neurofeedback protocol for migraine emphasizes protocols rewarding 12-15 HZ at the temporal lobes at sites T3 and T4 [22]. Siniatchkin and colleagues demonstrated a significant reduction in migraines in 10 young migraineurs after 10 sessions of neurofeedback at midline frontal and central areas teaching them to control slow cortical potential activity representing cortical sensitivity and reactivity [7]. Michael Tansey enabled four migraineurs to eliminate their migraines after neurofeedback training along midline frontal and central areas which showed that low frequencies became less dominant and higher frequencies were augmented [8]. An older study found that thermal biofeedback was no more effective than EEG alpha biofeedback and self hypnosis in treating migraine [9].

Neurofeedback training also includes a newer method called hemoencephalography, which targets the frontal lobe [23]. Passive infrared hemoencephalography (pIR HEG) is a form of biofeedback for the brain that measures and feeds back information on the thermal output of the frontal lobe [10,23]. Unlike electromyographic (EMG) feedback which involves lowering the tension of the frontalis or trapezius muscles, pIR HEG involves increasing the forehead temperature by watching a movie for feedback. The movie is in operation when the measured forehead temperature rises and the movie stops when the temperature drops. The therapist will increase the threshold as the client learns how to raise their forehead temperature. Clients are instructed to calmly concentrate on making the movie continue to play. Increases in the pIR HEG signal reflect a composite of thermal activity generated by vascular supply, vascular return and brain cell activity. 100 International Headache Society (IHS)-diagnosed migraineurs reduced the frequency of their headaches using this form of biofeedback [10,24,25].

## Methods

This is a single group outcome, open label study in a clinical setting where both the patients and those administering treatment were aware of the treatment being given. Patients were given Informed Consent for biofeedback methods administered as well as Informed Consent to Research as put forth by the lead author's ethics committee of the American Psychological Association and the Association of Applied Psychophysiology and Biofeedback. All signed copies remain on file at The Better Brain Center. The authors had full access to all the data in the study and take responsibility for the integrity of the data and the accuracy of the data analysis.

### Participants

Patients were recruited from the neurofeedback clinic of the Better Brain Center (formerly called Neurofeedback Consultants) where most were referred by their primary care physician or neurologist. Others responded to local media events covering their neurofeedback work in treating migraines. 74 headache clients presented to the clinic between 2004 and 2007. Selection criteria required that the client have migraine with or without aura and that this diagnosis be confirmed according to the IHS classification criteria for headache disorders [24,25]. Patients who had less than one migraine per month or more than 20 per month were excluded from the study. This broad range of 1 to 20 migraines per month was used to keep as many patients as possible in the sample; however, we also conducted analyses using just the subset of patients with 2 to 14 migraines per month, the more typical headache frequency criterion used in migraine studies. The total sample included 37 migraine patients (29 females and 8 males). Ages ranged from 9 to 79, with the majority (56%) between the ages of 16 and 52, and the remainder evenly split between the younger group (22% were between 9 and 15) and the older group (22% were between 55 and 79). In terms of medical history, most patients had long, stable histories of migraine and had tried multiple pharmaceutical treatments prior to neurotherapy. All were having at least one migraine per month and taking at least one type of medication (preventive, abortive or rescue) for their migraines and were not required to discontinue these during the study (See Table 1). About one-third of the patients had migraine with aura, and about three-fourths reported experiencing other kinds of headaches or one or more other significant conditions (e.g., anxiety, depression, problems with sleep or focus). All received a diagnosis of migraine or mixed migraine with tension type headache. Patients were screened for medication overuse headache and those taking abortive or rescue medications more than 2 or 3 times a week were referred to their prescribing physician for instructions on tapering down and possible alternatives to these types of medications.

**Table 1:** Number of patients on each type of migraine medication

Preventives	Preventives (off label)	Abortives	Rescue
Topamax- 5	Skelaxin- 1	Frova - 3	Alleve- 2

Depakote- 4	Requip- 2	Zomig- 2	Tylenol- 7

Inderal- 1	Cymbalta-3	Imitrex- 4	Phenergan-1

	Adderal- 3	Relpax- 3	Vicodin- 1

	Neurontin- 3	Maxalt- 1	Vicophren-1

	Lexapro- 2	Midrin- 1	Advil- 6

	Elavil- 1	Migrainol- 1	Fioricet- 4

	Botox- 1		Excedrin- 1

	Effexor- 4		Toradol- 1

	Verapamil- 1		Compazine-1

	Prednisone- 2		Butorphenol- 1

	Phenobarbitol- 1		

	Tegretol- 1		

	Atenolol- 1		

	Claritin- 1		

	Vasotec- 1		

	Tenex- 1		

### Initial assessments

A personal and family headache history was taken at initial evaluation and a diagnostic interview was performed by a licensed psychologist to confirm the IHS-diagnosis of migraine with or without aura and to assess other symptoms and conditions. All patients had also received a diagnosis of migraine by a physician (neurologist, family practitioner or OB GYN) prior to entering this study. For patients who did not have at least two weeks of headache diaries, they were asked to wait two weeks to begin treatment in order to keep a baseline daily diary to record headache frequency and severity. At the first session and every 10 sessions thereafter, clients were asked to complete a non-standardized checklist to indicate changes in headaches as well as other symptoms (e.g., anxiety, insomnia, other pain types, depression, and behavioral problems). These checklists, daily headache diaries and clinical interviews were used throughout treatment to help determine most effective protocols and placements which were modified accordingly.

### Follow up data collection

The data reported in this study were collected 3 months to 2 years after patients stopped coming to the neurotherapy center, either because they had completed the recommended number of treatment sessions or because they discontinued treatment on their own. The data were collected through follow-up telephone surveys conducted by a research consulting firm not affiliated with The Better Brain Center. Interviewers introduced themselves and indicated that they were conducting a follow-up assessment of The Better Brain Center's migraine patients for research purposes, and asked for permission to continue. Prior to starting treatment patients had been informed that their data may be used in a future retrospective study and that they might be interviewed for a study and all had agreed in writing. The telephone surveys were conducted during the last half of 2007 using a standardized protocol and prepared list of questions. The large majority of patients had completed treatment at least 6 months prior to the follow-up call, some as long as two years earlier. When asked about their post-treatment migraine history, participants were instructed to think about the 6 months immediately preceding the follow-up interview (not the entire time since their last treatment) and to estimate, on average, how many migraines they experienced per month. We included a broad range of months instead of just the 1 or 2 preceding months for two reasons. First, many patients experienced fewer than 1 per month post-treatment and a six month period is more likely to capture the fact that they are not completely migraine free. Second, many patients in this sample experienced fluctuations in the number of migraines they experienced per month, and capturing data for only 1 or 2 months could misrepresent their typical or average migraine frequency (although this is not a problem in large samples where unusually high or low frequency months should be randomly distributed across the sample; in smaller samples this is less likely to occur and short data collection periods can be an unnecessary source of error). Pre treatment headache frequency data was provided by headache diaries kept prior to treatment. Pretreatment headache frequency was also confirmed by interviews during follow up data collection. When asked about their migraine pattern prior to treatment, participants were also asked to recall the average number of migraines per month they were experiencing in the 6 months prior to seeking treatment. Interviewers had access to the migraine frequency data reported at the initial diagnostic evaluation, and if there were large discrepancies asked for clarification. Large discrepancies were rare, and were usually the result of misunderstanding the question or mixing headache types. In the 3 cases where participants could not be contacted or chose not to complete the telephone interview, migraine frequency data from patient records was used instead of the telephone survey data. In addition to the migraine frequency questions, we presented each respondent with a list of symptoms that are common among clients seeking neurotherapy (e.g., anxiety, focus or attention, depression, other (non-migraine) headaches, and asked which they had also experienced prior to treatment. For each symptom they reported having experienced we asked them to rate the level of improvement they experienced with treatment. The options for the 5 point rating scale included: 0 - No (0%) improvement, 1 Slight (10-30%) improvement, 2-Moderate (40-60%) improvement, 3-Major (70-90%) improvement, 4- Total (90-100%) improvement. We also asked all participants to use the same scale to rate the improvement they felt they had experienced in their migraines.

### Treatment protocol

The study involved treatment using EEG biofeedback, pIR HEG biofeedback and handwarming biofeedback for an average total of 40 sessions. Average length of time in treatment was 6 months. Subjects underwent an average of 30 frequency-based neurofeedback sessions and 10 pIR HEG sessions for 30 minutes at least twice weekly. Eleven patients had an interruption in their treatment after the initial 20 sessions of up to several weeks but returned for their remaining sessions.

Assessment: A neurophysiological assessment using EEG measurements was administered using two channels of the EEG amplifier/software Brainmaster Atlantis version 3.5 or Brainmaster version 2.5. EEG Data was collected in three minute segments, 2 channels at a time. Data was collected under eyes open, eyes closed and eyes open task conditions at each site. Ten sites were collected: frontal (F3, FZ, F4), temporal T3, T4) central (C3, CZ and C4) and parietal areas (P3, P4) using the International 10-20 system of electrode placement.

Data collected was used to determine peak amplitudes for specific frequencies within the 1-38 HZ range. Data collection was performed at each site with the ground at FZ and with ipsilateral references and with the ground at C3 when sampling the FZ site. Sampling rate was 256 samples per second using a third order filter and a 60 HZ notch filter. Amplitude measures were peak to peak magnitude.

Treatment: The EEG measures were used to guide neurofeedback training protocols by targeting frequency ranges with the highest amplitude. All migraine patients were trained to reduce the amplitude of the targeted frequencies. The EEG training primarily occurred at 5 sets of homologous sites - (T3-T4, C3-C4, F3-F4, FP1-FP2 and P3-P4). These homologous sites were chosen according to the lead author's training in neurofeedback in which years of clinical experience in treating migraines by other experienced clinicians is taught [22]. Electrode placements at homologous sites were used and training always began as a single channel placement using the first site as the signal and the second site as reference (example: T3-T4).

When training each site, each client received positive feedback (visual and auditory rewards via a video type of game) whenever EEG activity exceeded certain amplitude thresholds within a target band (called the reward band). Training each homologous site rewards the difference between the amplitude at each site of the target frequency. Simultaneously, clients received no feedback whenever excessive high amplitude activity occurred in certain frequency bands (called the inhibit band) that had been identified during the assessment. This lack of auditory reward (no beep or feedback) is designed to discourage the client from making excessive activity in the inhibit band whenever this activity exceeds the set threshold.

These protocols also reflect findings of EEG abnormalities commonly found in migraine patients [7,16-20]. The reward and inhibit frequencies chosen for each site were also selected according to the individual's neurophysiological assessment and they were also based on a history of training recommendations considered to be optimal for migraine stabilization by the authors and other practitioners in the field [22]. For example, for temporal lobe sites we rewarded a target range of 12-15 HZ. Lower target range rewards were selected at posterior and frontal regions. During training, sometimes reward frequencies were lowered by 2 HZ from the starting point. For T3-T4, for example, after starting at 12-15 HZ, we may move down to 10-13 HZ. Small changes in the targeted reward frequency can produce noticeable clinical improvement, readily identified by the migraine sufferer. For the migraine population, however, we have observed that lowering the target frequency further than 2 HZ frequently can have a destabilizing effect and may actually trigger a migraine.

Training Objectives: Patients were told simply to "make beeps" or an auditory reward which occurs when they achieve the training objectives of increasing the reward amplitude and reducing excessive amplitude in the targeted inhibit bands. The frequency ranges that were inhibited at all the sites ranged between 2 and 14 HZ (low inhibit) and between 15 and 38 HZ (high inhibit). The reward frequency range across all the sites was usually between 8-18 HZ, with a 3 HZ band width. Each set of sites had their own set of frequencies to be rewarded and inhibited and this stayed the same for each pair of sites for each individual patient.

Sessions commenced at the temporal locations (T3, T4) for three to five sessions and then moved to central areas (C3, C4), then frontal (F3, F4), prefrontal (FP1, FP2), and parietal (P3, P4) for typically one to two sessions at each location. Only these five pairs of sites were used with the migraine protocols. We find that starting at the temporal lobe locations seems to offer the most powerful relaxation and stabilization initially in order for us to be able to proceed to the other locations which may not always be as relaxing or stabilizing. If patients had an adverse reaction to any of these sites, we moved on to another location. On rare occasions, a patient would respond by becoming too activated by work at a certain site so we would move to other sites in the treatment regimen. A typical treatment regimen would resemble the following: Sessions 1-4- EEG biofeedback at the temporal lobes simultaneously with thermal handwarming biofeedback, session 5-9- EEG biofeedback at central, frontal and parietal areas simultaneously with thermal handwarming biofeedback, Session 10, 12, 14, 16, 18, 22, 26, 30, 34, 38- pIR HEG biofeedback, Sessions 11,13, 15,17, 19-21, 23-25, 27-29, 31-33, 35-37, 39 & 40- EEG biofeedback at various locations along the cortex (depending on patient presentation and response) simultaneously with thermal handwarming biofeedback. Although we do only one or two sites per session, we have found the best responses with migraineurs when we continue to move around the whole head at various placements because we find that this population can be easily irritated by the overtraining of one site.

For most patients 30 minutes of pIR HEG biofeedback was introduced at approximately their tenth visit. This involved the patients wearing a headset which is designed to be worn at FPZ (center of forehead) and watching a movie and being challenged to keep the movie playing as the reward threshold was re-set to higher temperatures. The movie would remain playing as long as the patient's forehead temperature increased as the threshold was re-set. After two sessions the frequency-based neurofeedback training was reintroduced, and generally conducted pIR HEG every 2^nd ^or 3^rd ^session along with neurofeedback for the remainder of the treatment period. Rationale for changing the order and number of each of these protocols was based on patient tolerance and effectiveness of each protocol and was always subject to change based on patient feedback. We have found that if we allow the patient to elevate their forehead temperature more than 2 degrees, or if they are worked too aggressively, it is more likely that this technique can have the side effect of causing rather than aborting a migraine. Therefore, patients were asked to gently but mindfully increase their temperatures in order to watch the movie and we did not challenge them aggressively to do so by too frequently resetting the reward threshold.

Thermal handwarming biofeedback was also used simultaneously along with the EEG biofeedback during clinic sessions. All patients were given thermal biofeedback units along with instructions for how to perform thermal biofeedback at home on the days they did not have a clinic session. An analysis of biofeedback in combination with home training was found to be more effective than therapies without home training [5]. The objectives of this study are to show that the combination of both neurofeedback modalities along with thermal biofeedback and medication will significantly lessen the frequency of headaches experienced by the participants and that these effects will be more robust than thermal biofeedback-only approaches and more effective and enduring than traditional medication-only approaches.

### Instrumentation

Frequency-based EEG biofeedback protocols used the Neurocybernetics (EEG Spectrum, International, Canoga Park, CA) with Procomp amplifiers (Thought Technology, Montreal, Quebec, Canada) or the Brainmaster systems (Brainmaster Technologies, Oakwood Village, OH). Passive Infrared Hemoencephalography units were also used (Jeffrey Carmen, Manlius, NY). Thermal handwarming biofeedback utilized the SC-911 unit (Biomedical Instruments, Inc., Warren, MI).

## Results

Table 2 shows the age and gender of everyone in the full sample and the pre-treatment and post-treatment migraine frequency estimates. The estimates are based on participant reports of the average number of migraines they experienced per month in the 6 months prior to treatment, and the 6 months immediately preceding the follow-up telephone survey. The small number of participants (n = 7) who had completed treatment only 1 to 5 months before the follow-up interview, reported migraine frequency for this shorter post-treatment time period. The pre-treatment mean frequency was 7.6 migraines per month (S.D = 5.1) the post-treatment mean was 2.9 migraines per month (S.D = 2.8), and the mean difference was 4.72 (S.D. = 4,32) few migraines per month. The standardized effect size (derived by dividing the mean difference score by the standard deviation of the difference scores) is 1.09, an effect size considered in the literature to be very large. Since many migraine studies include only those who experience 2 to 14 migraines per month we also calculated effect size eliminating the 5 patients with 15 to 20 migraines/month and the 4 who experienced only one per month. This produced and even larger effect size: 1.23. We next added in 3 "dummy" cases showing no change to address concerns that those who fail to complete the minimum number of sessions (the minimum was 20, most had at least 40) might have been non-responders. This effort to approximate an " intention to treat" analysis, assuming a 10% non-completer rate, reduced the 1.23 effect size in the restricted , 2 to 14 migraine sample, to 1.00, still a very large effect size.

**Table 2:** Sample characteristics and average number of migraines per month pre- and post-treatment

Age	Gender	Average # of migraines/mo pre-treatment	Average # of migraines/mo post-treatment	Difference
40	f	1	0	1

41	m	1	0	1

9	m	1	0.16	0.84

15	f	1	0	0.7

50	f	2	0.5	1.5

39	f	2	2	0

20	m	2	0.3	1.7

42	f	3	3	0

62	f	3	0	3

9	m	3	1	2

52	f	4	4	0

57	f	4	0	4

13	f	4	0.5	3.5

14	f	5	3	2

27	m	6	6	0

10	f	6	4	2

33	f	6	1	5

47	f	7	5.5	1.5

21	f	8	0.5	7.5

69	f	8	8	0

18	f	8	2	6

25	f	8	0	8

38	f	9	0	9

58	f	10	4	6

16	f	10	10	0

49	f	10	2	8

44	f	10	3	7

45	f	10	1	9

48	m	10	6	4

79	f	10	4	6

55	f	13	10	3

15	m	14	5	9

55	f	15	4	11

59	f	15	5	10

15	f	15	3	12.5

20	f	18	2	16

47	m	20	7	13

**Figure 1:**
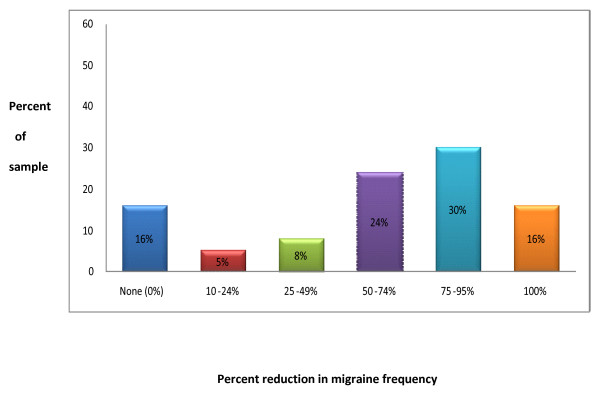
**Percent of total sample who experienced levels of improvement in migraine frequency**.

For each individual we also calculated the percent reduction in migraine frequency by dividing the difference between that individual's pre- and post-treatment migraine frequency estimates by the average number of pre-treatment migraines they experienced. As illustrated in Figure 1, 70% of the sample (or 26/37) showed a 50% or greater reduction in the frequency of their migraines, and only 16% (or 6/37) failed to improve at all.

The significance of the observed changes was examined using the Wilcoxon signed ranks test, a non-parametric alternative to the t-test for small sample studies where the dependent variable is not normally distributed. In the Wilcoxon signed ranks test the differences between pre- and post-treatment scores are rank ordered, and the significance test is based on ranks, eliminating the potential biasing effects of large, spurious differences in either direction. If the treatment has no effect the sum of the ranks where the difference is positive should be nearly equal to the sum of the ranks where the difference is negative.

In the present case, there was a large difference; in 31 cases post-treatment scores (average number of migraines per month) were less than pre-treatment scores, in 6 cases scores were equivalent, and there were no cases where post-treatment scores were greater than pre-treatment scores. The resulting z-score of -4.86 was statistically significant at the p < .001 level.

Although the focus of this study was on migraine headaches, patients seeking neurotherapy are typically experiencing more than one problem, and migraine patients are no exception. In the follow-up interviews we asked participants to (a) indicate which of several other common symptoms they were experiencing when they first sought treatment, and then (b) use a 5-point scale to rate the level of improvement they experienced following neurotherapy treatment. The response scale options were "no improvement" (0), "slight (10-30%) improvement" (1), "moderate (40%-60%) improvement" (2), "major (70-90%) improvement" (3), and "total (90-100%) improvement" (4). Table 3 shows the number of individuals rating the 6 most common symptoms (migraine is included and the N of 34 indicates that we did not get ratings from 3 of the migraineurs who provided headache frequency data), and the percent reporting three levels of improvement. The first group includes those who selected either the "No improvement" or the "Slight (10-30%) improvement" response options, the middle group includes those who selected the "Moderate (40-60%) response option, and the third group includes those who selected "Major (70-90%) or Total (100%) improvement. Migraines were the most improved symptom based on this scale, with 62% or 23/37 reporting major or total improvement, followed by "other headaches," where 50% or 19/37 reported major or total improvement. The percent reporting major or total improvement on other symptoms ranged from 32% to 41%. Sleep problems were least likely to be substantially improved.

**Table 3:** Ratings of improvement on migraine and other presenting problems

	Number giving ratings	Slight or no improvement	Moderate improvement	Major or total improvement
Migraine headaches	34	21%	18%	62%
Anxiety	31	32%	36%	32%
Focus	29	35%	28%	38%
Other headaches	28	25%	25%	50%
Depression	27	33%	26%	41%
Sleep	22	46%	23%	32%

## Discussion

The concept of an under or overaroused nervous system was first proposed by Nobel Laureate Walter Rudolph Hess who in the 1950s experimented with electrical stimulation of the brain which led to changes in arousal [26]. It has been theorized that disorders of attention, affect and pain are due either to over or underaroused brain states, and that neurofeedback is effective for a variety of symptoms or symptom clusters because it improves the brain's ability to regulate these arousal states [13]. Neurofeedback treatment protocols address the underlying arousal problem, obviating separate validation studies for every medical diagnosis [13]. In this study, it appears that the biofeedback enabled the patients to gradually learn to control their susceptibility to getting headaches. Generally, they began to notice gradual improvements early on in treatment, particularly in their ability to manage stress, which was impetus for continuing treatment. This was assessed every 10 sessions by a written checklist and by interviews with a psychologist at each session. By session 20, most began to be aware of their ability to control or prevent their headaches. In most cases, by session 40, patients felt a sense of increased mastery over being better able to recognize when they were at risk (increased autonomic arousal in reaction to stress) and to take appropriate measures to be able to prevent headaches. 40 sessions happened to be the average number of sessions undertaken in the study. Number of sessions ranged from 20-67 and was determined by what treatment provider and each patient felt they needed in order to ultimately learn to control migraines. These patients described the biofeedback as helping them to acquire the ability to better self-regulate by learning to control their EEG and reducing muscle tension, slowing the rate of their breathing and warming their hands and forehead, all of which were necessary for the types of biofeedback they had undergone. When asked how they thought they were better able to prevent headaches during interviews at each session and on checklists after every 10 sessions, many would explain that during potentially stressful conditions they would imagine hearing or visualizing the neurofeedback games and this appeared to help them invoke the physiological state elicited during the actual sessions. We have observed that thermal biofeedback devices (pIR HEG machine and the handwarming units) can often be powerful migraine abortives once patients learn to raise their hand or forehead temperatures. All clients, whether or not they were successful at reducing their migraines, demonstrated an ability to warm their hands and foreheads and decrease their elevated EEG amplitudes of both slow and fast-wave activity. Patients related during session interviews that these techniques have eventually enabled them to automatically learn to abort their headaches without having to use the actual devices. Of the 37 patients in the study, five had fifteen or more migraines a month and all five improved significantly which may show promise that these methods can be useful for preventing the progression from episodic to chronic migraine.

Central nervous system dysfunction may play a key role in the pathogenesis of migraine [16-21]. As there are no apparent structural disturbances, clinical neurophysiological methods may be well-suited to study its pathophysiology [16]. In both migraine with and without aura, somatosensory evoked potential studies show that lack of habituation in cortical information processing between attacks is a reproducible central nervous system abnormality with this population [19]. Siniatchkin et al demonstrated the vulnerability of the migraine brain by measuring the effects of experimentally-induced stress on the contingent negative variation (CNV) response, which is a slow cortical potential believed to reflect altered excitability. This study showed a susceptibility to stress-induced migraine provoking agents before an actual attack [20]. Additionally, it has been observed that abnormal behavioral patterns such as hypersensitivity and perfectionism are often characteristic among migraine sufferers yet these psychological features may be the result of an innate cortical hypersensitivity in addition to associated social learning processes [21]. In this study neurofeedback appears to have improved stress resilience and susceptibility to migraines in the migraine participants. This may be due to the increase in self regulation brought about by the process of long term potentiation that may result from the operant conditioning of the EEG during the neurofeedback training [12].

Migraine has a comorbid association with a number of psychiatric conditions, including bipolar disorder, anxiety states, and depression, all of which are associated with perturbations in the serotonin and norepinephrine neurotransmitter substances [27,28]. Depression is often comorbid with migraines and anti-depressants are often used to treat both conditions [29]. Evidence that many neurological conditions are comorbid and alleviated by identical or very similar drugs supports three important principles in the spectrum paradigm: a) different symptoms are often manifestations of the same underlying instability or in balance, b) symptoms manifest differently depending on where they fall along the continuum of the underlying dysfunction, c) treatments need not be "disease specific" to be helpful [13]. Neurologist Oliver Sacks' speculation that brainwave biofeedback might prove useful for migraines after showing promise in treating seizures supports the spectrum concept of related disorders responding to one mode of treatment [30].

Migraine and tension type headache were linked after both types showed a significant response to sumatriptan. A convergence hypothesis was proposed speculating that the entire clinical spectrum of headache may share a common physiological pathway based on one type of medication exerting an effect on two distinctly different types of headache [31,32]. Similarly, an older study shows that neurofeedback was effective for tension type headache [11] and our study finds that several types of biofeedback have an effect on migraines, other types of headache and other comorbidities.

Biofeedback used with medications appears to outperform medications alone [5,33,34]. In our study involving biofeedback with clients using medications, we saw the frequency of usage of the abortive and rescue medications drop along with the frequency of headaches.

## Limitations

This study did not focus solely on neurofeedback as a stand-alone treatment. The simultaneous inclusion of the peripheral biofeedback treatment modalities already known to be effective for migraines confounds this study. The study design is not ideal in that it prevents any supposition as to which interventions may be at work in cases of improved headaches. Because the study was unblinded and the treatment protocol was modified according to individual patient reactions, it does not meet the standards for a clinical trial. A single group (non-random) retrospective review of a convenience sample of headache sufferers without a control group and interviewed as long as 24 months after treatment with a varying treatment protocol is perhaps reflective of clinical practice but not ideal for investigating a novel approach to managing headaches. Additionally, the response scales used to construct Table 3 did not include the choice of "worse", which may have induced an overly acquiescent response bias. The simultaneous measuring of pre and follow-up data is problematic also, since it may introduce several potential biases such as memory and recency effects.

Great care was also taken to rule out positive biases that might have been introduced by having data collected by people the patients had known or seen at the clinic. Those conducting the telephone survey made an effort to assure respondents that this was an objective effort to learn about both positive and negative outcomes. Nevertheless, retrospective reports of migraine frequency are not ideal. If we were paying subjects to participate in an externally funded study, we could require participants to complete daily headache diaries for several months post-treatment. This is not practical in practice-based research, however, especially when treatment has effectively reduced the frequency or severity migraines to the point that they are no longer a focus of concern.

## Conclusions

Migraine may be progressive disorder with an excellent response to preventive early interventions [33,34]. Yet none of the pharmaceutical options are exceptionally effective or without side effects. The best result that medication has achieved has been only about a 50% reduction in approximately 50% of migraine patients [34]. Our study outperforms this by achieving a 50% or more reduction in 70% of the participants based on follow-up data collected on average, 14 months after patients had completed at least the minimum recommended 20 treatment sessions. The treatment effect sizes we obtained (1.09) are greater than those reported in a recent meta-analysis for either EEG-biofeedback (about .4) or temperature training feedback (about .5) or blood volume pulse feedback (about .7) alone, or temperature feedback plus electromyographic feedback (about .6) [5]. Although we did not have a control group, and thus cannot completely rule out placebo effects in this study, it may be unusual for a placebo effect to last 6 months to two years.

Despite the different types of intervention used in our study (manipulation of the EEG, forehead temperature or hand temperature), the retrospective reports of migraine frequency and the absence of a control group, the statistically and clinically significant improvements observed in this patient population attests to the promise biofeedback based treatment modalities hold for migraine patients. It is our hope that this study will generate an interest in performing larger scale controlled studies in the non-invasive neurotherapies to treat migraine and other chronic and/or progressive disorders.

## Abbreviations

EEG: electroencephalogram; pIR HEG: passive infrared hemoencephalography; qEEG: quantitative EEG; CNV: contingent negative variation.

## Competing interests

The authors declare that they have no competing interests.

## Authors' contributions

DAS designed the study, carried out the treatment, initiated the study, performed the research and wrote this article except for most of the methods, results and conclusions sections. MSL participated in the study design, oversaw the data collection, performed the statistical analysis, supervised the data analysis and wrote most of the methods, results and conclusions sections. All authors read and approved the final manuscript.
